# A Comparison of Frameworks Evaluating Evidence for Global Health Interventions

**DOI:** 10.1371/journal.pmed.1001469

**Published:** 2013-07-09

**Authors:** Jill Luoto, Margaret A. Maglione, Breanne Johnsen, Christine Chang, Elizabeth S. Higgs, Tanja Perry, Paul G. Shekelle

**Affiliations:** 1 Southern California Evidence Based Practice Center, Rand Health, Santa Monica, California, United States of America; 2 Agency for Healthcare Research and Quality, Rockville, Maryland, United States of America; 3 National Institute of Allergy and Infectious Diseases, National Institutes of Health, Bethesda, Maryland, United States of America; 4 West Los Angeles VA Medical Center, Los Angeles, California, United States of America

## Abstract

Jill Luoto and colleagues apply different frameworks to the same body of evidence for three advocated global health interventions and compare the ratings and policy recommendations resulting from each.

*Please see later in the article for the Editors' Summary*

**Summary Points**

- Evidence-based decision-making is critical to informing policy in global health interventions and programs.
- Existing frameworks for evaluating evidence that were developed or recommended for community or public health decision-making vary in their criteria and application.
- We compared how different community or public health evidence frameworks assessed the same body of evidence for three advocated global health interventions and find there can be substantial differences in the rating of evidence, which could contribute to differences in policy recommendations.
- All current frameworks emphasize effectiveness, and have shortcomings on other important factors into policy decision-making such as costs, implementation issues, context, and sustainability.
- As global health policymakers move towards evidence-based approaches, we find a gap between what is currently available and the needs for an evidence framework appropriate for application to a global health setting in a low- and middle-income country context. More work is needed to either adapt one or more existing frameworks, or to develop an entirely new framework to meet the needs of policymakers and others responsible for implementing global health interventions.

## Introduction

A major movement in global health and development in the past 10 years has been the enthusiastic adoption by many of randomized controlled trials (RCTs) from the field of medicine to represent the most rigorous method to evaluate a program's causal impact [1]–[4]. More recently, this movement has brought about a conceptual debate in global health and development about the proper role for RCTs in informing policy, with increasing efforts to “mind the gap” [5] between the evidence generated by RCTs (which focus on internal consistency) and the larger policy questions at the level of communities or populations (which require, among other things, generalizability) [4],[6]–[10]. The field of medicine that developed the RCT also developed the concept of “evidence-based” medicine that aims to improve health policy decision making by encouraging policymakers to base their policies on the best available evidence. Large international policy-making bodies appear set on applying a similar concept to global health and health systems research [4],[11]. In order to be evidence-based, decisions about global health interventions must consider the available evidence in terms of its quantity, quality, and relevance. Rather than use implicit judgment or other ad hoc methods, in evidence-based medicine it is now advocated and common practice to use a formal framework for considering the evidence as part of a systematic review, the advantages of which include increased transparency and better decision-making. Formal frameworks for evaluating evidence about community-level public health interventions have been proposed and advocated for similar reasons [12]–[17]. These frameworks differ in the degree to which they weight the importance of data from RCTs as compared to data from other study designs, the magnitude of potential benefits and harms, the role of context and implementation, and other factors. At present, there are no commonly accepted guidelines within global public health for how to evaluate evidence, and there is scant evidence to guide policymakers when selecting a framework to use for assessing a body of evidence about a global health intervention. We sought to assess how summary conclusions about the evidence for interventions or programs currently in use or proposed for wide adoption could be influenced by the choice of framework. Consistent results across frameworks would increase policymakers' confidence in using and applying evidence frameworks, and may thereby help to narrow the gap between the questions asked by global health researchers and policymakers. Inconsistent results would call for a re-examination of current frameworks in terms of the domains they assess and the ways in which they are applied.

## Identifying and Applying Evidence Frameworks to Support Policy Decision Making

We define a global health evidence framework as one which uses multiple domains to arrive at a summary judgment of the evidence for community or population health interventions or programs, which could be applied to the kinds of interventions or programs that are commonly being considered in low- and middle-income countries. This includes frameworks explicitly developed for global health interventions, frameworks that were presented with a global health intervention as an illustrative example of its application, and general community or population health frameworks that could be applied to global health interventions. Details of our search methodology are summarized in Box S1, from which we identified six frameworks [12]–[17]. Table 1 lists some key characteristics of each framework. Although our search methods were extensive, it is possible there are additional frameworks that we did not identify. However, the frameworks we did identify are a sufficient sample to explore the issue of whether potential policy recommendations derived from use of a framework could be sensitive to the choice of framework. All six frameworks indicated that their goal was “grading” (or “evaluating”) “evidence” on “interventions.”

**Table 1:** Summary of existing public health frameworks considered.

Framework name	Grades Assigned/What the Framework Rates	Domains for Grading Evidence	Notes on Domains
**Tang et al. ** [**12**] (Drawn from article's Table 1 for grading of evidence on association, repeatability and causal mechanism)	Grades of 1, strong; 2, weak; 3, insufficient. Expanded categories include 2A, probable; 2B, possible; and 2C, limited.*“Grading of evidence of the effectiveness of health promotion interventions.”*	Association	“High” association is defined as a RR of greater than 2. Otherwise “low” or “none.”
		Repeatability	Wide or limited
		How it works	How it works is known or not known
**GRADE ** [**13**] (Summarized from article's Table 1.)	Four grades assigned: high, moderate, low, and very low quality of evidence.*“A system for rating quality of evidence/confidence in estimates of treatment effects.”*	Randomized trials start with a “high” initial quality grade, observational studies start with a “low” grade.	Grades can be moved down depending on factors such as risk of bias or inconsistency, or up in light of a large measured effect or evidence of a dose-response.
**HASTE ** [**14**]	Four grades assigned: 1, strong; 2, conditional; 3, insufficient; 4, inappropriate. Grade 2, conditional, has subcategories of probable, possible, and pending.*“A novel system of evaluating evidence for interventions targeting decreasing HIV risk specifically among most at risk populations.”*	Efficacy	Whether consistent, limited or inconsistent
		Biological plausibility	
		Implementation data availability	Whether available or not
**USCPSTF ** [**15**]	Evidence is characterized as strong, sufficient, or insufficient.*“Evaluate and make recommendations on population-based and public health interventions”…a “process to systematically review evidence and translate that evidence into recommendations.”*	Execution	Good or fair
		Design suitability	Greatest (RCTs), moderate (no concurrent comparison group), or least
		Number of studies	
		Consistent	“Generally consistent in direction and size”
		Effect size	Sufficient or large, defined on a case-by-case basis based on Task Force opinion
		Expert opinion	Whether used or not
**NHMRC ** [**16**]	Four grades assigned: A, excellent; B, good; C, satisfactory; D, poor. Grade A can be trusted to guide practice; grade D concludes the body of evidence is weak and recommendation must be applied with caution.*“A new approach to grading evidence recommendations, which should be relevant to any clinical guideline (not just those dealing with interventions).”*	Evidence base	“Evidence hierarchy” places systematic reviews of RCTs with “low risk of bias” highest
		Consistency of evidence	
		Clinical impact	Very large, substantial, moderate, slight
		Generalizability	Highest grade awarded if “population/s studied in body of evidence are *the same* as the target population for the guideline” (emphasis added)
		Applicability	
**NHS Health Development Agency ** [**17**]	Four grades assigned: A, B, C, and D.*“This provisional framework provides a practical and transparent method for deriving grades of recommendation for public health interventions, based on a synthesis of all relevant supporting evidence from research.”*	Efficacy	High quality meta-analyses and systematic reviews of RCTs with very low risk of bias rated highest level of evidence.
		Evidence of corroboration	Strong evidence of corroboration defined as “Consistent findings in two or more studies of ++ quality carried out within the UK and applicable to the target population, providing evidence on salience and implementation.” ++ is defined as is efficacy above.

We next identified a diverse set of global health interventions as potential candidates with which to apply these existing frameworks by considering the major causes of morbidity and mortality in developing countries or the major diseases of focus among international global health financing bodies. We developed a draft set of key dimensions for classifying global health interventions in order to map out these potential exemplars to select a diverse set of interventions along these dimensions (e.g., population affected, whether the intervention addresses a communicable or non-communicable disease, etc.). We were advised on this project by a multidisciplinary panel of experts (listed in Acknowledgments) composed of global health experts in academia, donor agencies, policymakers, and practitioners who provided input on the dimensions and on their preferred exemplars. From this exercise, we selected three interventions as exemplars for assessing the frameworks that represented a diversity of interventions: household water chlorination, prevention of mother-to-child transmission of HIV (PMTCT), and lay or community health workers to reduce childhood morbidity and mortality. Table S1 demonstrates the diversity of these exemplars across our identified dimensions, and Box S2 presents the full list of potential exemplars from which these three were chosen.

For each of the three chosen global health exemplar interventions we located published systematic reviews of their effectiveness by conducting a Medline search. For each of these reviews, we retrieved the original research studies cited and used both the original studies and the systematic reviews as sources of evidence when applying the frameworks. As is customary and recommended in most evidence-based medicine processes, we used two members of the research team to independently apply the six frameworks to this evidence base for each of the three exemplar interventions. Disagreements were settled by a group consensus process. The results of the applications were compared both quantitatively (i.e., in how many cases was there congruence among frameworks) and qualitatively. Table S2 summarizes the evidence base for the three chosen global health exemplars, their primary outcomes of interest, and their associated systematic reviews and original research studies.

## Different Evidence Frameworks May Support Different Policy Decisions

Table 2 summarizes our findings from the application of the six evidence frameworks to the three global health exemplars. We focus our attention on a comparison of the summary conclusions for each outcome/exemplar using the different frameworks. More details for how we assigned grades to a particular outcome are available in an Agency for Healthcare Research and Quality report [18].

**Table 2:** Results on three exemplars applied to six evidence frameworks.

Exemplar	Outcomes	Tang et al. [12]	GRADE [13]	HASTE [14]	USCPSTF [15]	NHMRC [16]	NHS Health Development Agency [17]
**Household water chlorination**	Diarrhea	Grade 2b level 1 possible	⊕⊕⊕ Moderate quality of evidence	Grade 2b - Possible	Strong	“C” - Satisfactory	“B”
**Preventing mother-to-child transmission, all regimens included**	HIV infection in child within year of birth	Grade 2b level 1 possible^a^	⊕⊕⊕⊕ High quality of evidence	Grade 1- Strong	Strong	“A” - Excellent	“A”
**Lay health workers in primary or community health care to reduce morbidity in children under age 5, compared to usual care**	Morbidity in children under 5	Grade 2b level 2 possible^b^	⊕⊕ Low quality of evidence	Grade 3 - Insufficient	Strong	“B” - Good	“C”

a Tang et al. grade for PMTCT is due to strict rule that only interventions with relative risk (RR)>2 qualify as “strong” evidence. If this rule is flexible we would rate PMTCT as “Grade 1 Level 1 Strong” by Tang et al. categorizations.

b Grade 2c level 2 if repeatability outside Southeast Asia is not considered acceptable.

For studies of household water chlorination, we consider the primary clinical outcome of (self-reported) diarrheal incidence over measured water quality due to its clinical importance. The evidence frameworks generally conclude that the evidence for diarrheal outcomes is weak or moderate. Only the U.S. Community Preventive Services Task Force (USCPSTF) framework assigns household water chlorination its highest grade (“strong”). All of the remaining frameworks assign the evidence grades that are lower than their highest possible rating, with the evidence classifications ranging from the highest categorization of “strong” by the USCPSTF framework, to the next-to-lowest grade of “C – satisfactory” within the Australian NHMRC framework.

For PMTCT studies, all of the frameworks assign their highest possible grade to the body of evidence with the exception of the framework by Tang and colleagues, which assigns a “Grade 2B, Level 1 Possible.” However, this grade is the result of our strict interpretation of the rule that only interventions with a relative risk (RR) of greater than two qualify as “strong.” If there is some flexibility with this strict cutoff, the rating would change to the highest grade of “Grade 1 level 1 strong.”

For interventions involving community or lay health workers, we chose the outcome “reduce morbidity in children under 5 years old compared to usual care” as it seemed both to be an outcome very important to communities and to have enough studies to make a meta-analysis meaningful. With this intervention the various frameworks again generally rate the evidence as being of low or moderate quality with the exception of USCPSTF, which assigns the highest grade of “strong.” HASTE, on the other hand, would rate this same body of evidence as grade three “insufficient,” and GRADE also assigns it a “low quality of evidence.”

Overall, Table 2 shows that for two of the three exemplars assessed, at least one framework resulted in an overall assessment that varied by at least two categories from one or more of the other frameworks when applied to the same evidence base (i.e., from “A” to “C,” or from “strong” to “insufficient,” etc.).

## Discussion

We find that assessing the same body of evidence using existing public health frameworks yields somewhat to markedly different conclusions depending on the framework applied. Thus, in practice, if the current push towards evidence-based global health policy making includes adoption of an evidence framework (one key method for ensuring an “evidence-based” approach), the choice of framework for evaluating the evidence could potentially lead to different policy decisions, a potentially unintended consequence of the choice of framework. For example, had policymakers used the USCPSTF framework, they would have reached the conclusion that all three interventions were equally strong and supported. Conversely, had policymakers used the GRADE or HASTE framework, they would have concluded that the three interventions varied from “insufficient” or “low quality” to “strong” and “high quality.” Had six different policymakers been considering the same evidence on household water chlorination to reduce diarrheal outcomes and each used a different framework, they could have reached differing conclusions about the strength of support that ranged from grade “C” to grade “B” to “possible” to “moderate quality” to “strong.” Actual policy decisions will include other factors, such as feasibility, financial resources, and health systems capacity, but the current push for “evidence-based” decision-making makes the adoption of an evidence framework likely, and, therefore, the rating of evidence would likely be one important factor in decision-making.

Why should these frameworks differ in their conclusions? One possible reason is that they differ in whether and to what degree they deal with the following domains: (1) how strict or explicit the rules are for classifying the strength of evidence; (2) the magnitude of potential benefits versus harms; (3) what role, if any, context is taken into consideration in evaluating the evidence; (4) how much is reported about the details of implementation; (5) whether the ease of implementing the intervention or program is taken into consideration; (6) total costs for the program or intervention; and (7) sustainability of the program or intervention, both cost-wise and programmatically. The USCPSTF, Australian NHMRC, the UK National Health Service (NHS) Health Development Agency, and GRADE have stricter rules for classifying the strength of evidence than the HASTE framework and the framework from Tang and colleagues, which allow for more individual interpretation. The Tang and colleagues framework, GRADE, the USCPSTF, and Australian NHMRC all make explicit a consideration of the magnitude of the benefits, while HASTE and the NHS Health Development Agency do not. Only the Australian NHMRC framework explicitly considers context, and only the HASTE framework includes a detailed assessment of implementation data, although context could be considered part of “widely demonstrated” in the Tang and colleagues framework and could be considered in the “corroboration” criterion in the NHS Health Development Agency Framework. The USCPSTF considers barriers to implementation in their evidence review but not as part of the overall assessment of the body of evidence. Costs and sustainability are not included routinely in any of the frameworks, although GRADE does have guidance on including cost as an outcome and on incorporating cost into the strength of the evidence [13],[19], and the USCPSTF searches for cost information on recommended interventions. While it is likely that not all of these frameworks necessarily had as goals the assessment of information on costs, contexts, or implementation, it is important to note their absence because experts consider these to be crucial aspects of the assessment of evidence about global health interventions for policy decision-making. Their absence from the frameworks could be due to their original absence from the evidence base – that is, the published systematic reviews on the exemplars and the original articles included in those reviews, which also may not have had as their primary objective identifying evidence about implementation, cost, sustainability, etc. However, the absence of this kind of evidence from the reviews and the original articles included in them means that the evidence is also not generally available to policymakers who need to make decisions. This gap between the needs of health care policymakers and the research products of global health researchers is one that would likely need to be closed if global health policies are to be improved.

An additional cause for variability in the conclusions among different frameworks when assessing the same global health evidence may be variability in applying the individual frameworks themselves. When individual team members initially applied the frameworks to the evidence, they sometimes reached different conclusions, largely due to the need for individual interpretation of the criteria used in the frameworks. These differences were resolved in a consensus process, as is standard practice in most evidence-based medicine processes. Nevertheless, this situation raises the possibility of potentially poor inter-rater reliability within frameworks, which has also been observed with frameworks used to assess the risk of bias or strength of evidence for conventional medical therapies [20]–[22]. With our study design, it is not possible to estimate the relative contributions from these two potential contributing factors (the differences between frameworks in the domains to be considered and how they are scored versus poor inter-rater reliability) on our conclusions. However, we found that across raters, no initial grades differed by more than one category, whereas across systems we did find differences of two or more grades.

Although a similar exercise could have been undertaken with more than three exemplars, our initial choice of three proved sufficient to identify variability both within and across frameworks in how evidence is assessed. Moreover, additional exemplars will not change the identification of context, costs, and implementation data as important missing domains of these frameworks. We also recognize that our results may be sensitive to the composition of participants on our technical expert panel who provided input at each stage of this process, and further evaluation of these results with a wider group of stakeholders is warranted. However, these stakeholders' identification of a need for more data about implementation is consistent with the increasing recognition of the importance of implementation reporting in other health-related fields [6],[23],[24].

As global health policymakers move towards evidence-based approaches, our study reveals a gap between what is currently available and the needs for an evidence framework appropriate for application to a global health setting in a developing country context. More work is needed to either adapt one or more existing frameworks, or to develop an entirely new framework to meet stakeholders' needs. For example, Lewin and colleagues on the Task Force on Developing Health Systems Guidance of the World Health Organization recently described the beginnings of an adaptation of the GRADE framework [25]. Current frameworks for evaluating evidence on public health interventions have evolved from the clinical model where decision making is determined by rigorous systematic review of efficacy trials, usually based on data derived from RCTs that emphasize efficacy for the individual patient. Yet the evidence requirements for scaling up global health programs include three key elements: efficacy at the individual level, effectiveness at the population level, and sustainability at the host-country level. These evidence streams can often result from disparate research approaches, implying an additional set of needs when evaluating the evidence. A global health evidence evaluation framework must be systematic while being able to incorporate relevant information from studies on context or other details that are not traditionally reported in published findings from RCTs. We recommend that the global health community work to develop a framework or frameworks that can take into account evidence relevant to all three key elements needed for policy decision making, which can be applied with a reliability sufficient to give policymakers confidence that differences in ratings reflect differences in the underlying evidence. Such a framework could help to improve the flow of information between researchers and policymakers, as well as narrow the gap between them in terms of the questions they ask and the tools they utilize to answer them.

## Supporting Information

Box S1 **Search methodology.**
(DOC)

Box S2 **Full list of potential exemplars.**
(DOC)

Table S1 **Diversity of exemplar interventions across key dimensions.**
(DOC)

Table S2 **Evidence base for three global health exemplars.**
(DOC)

## Abbreviations

3IE: Impact Evaluation
CHWs: community or lay health workers
EVIPnet: Evidence Informed Policy Networks
GRADE: Grading of Recommendations Assessment, Development and Evaluation
HASTE: Highest Attainable STandard of Evidence
NHMRC: Australian National Health and Medical Research Council
PMTCT: Prevention of Mother-To-Child Transmission
RCTs: randomized controlled trials
RR: relative risk
SUPPORT: SUPporting POlicy relevant Reviews and Trials
SURE: Supporting the Use of Research Evidence for policy in African health systems
USCPSTF: U.S. Community Preventive Services Task Force
WHO: World Health Organization

## References

[1] Kremer M, Glennerster R (2011) Improving Health in Developing Countries: Evidence from Randomized Evaluations, Chapter 4. Handbook of Health Economics 201–315.

[2] Banerjee AV, Duflo E (2009) The Experimental Approach to Development Economics. Annu Rev Econom 1: 151–178.

[3] Banerjee A, Duflo E (2011) Poor Economics: A Radical Rethinking of the Way to Fight Global Poverty: Public Affairs. 320 pp.

[4] Yamey G, Feachem R (2011) Evidence-based policymaking in global health - the payoffs and pitfalls. Evid Based Med 16: 97–99. 21693456 10.1136/ebm.2011.100060

[5] (2011) Mind the Gap: From Evidence to Policy Impact. International Initiative for Impact Evaluation. Conference held in Cuernavaca, Mexico. Available: http://www.3ieimpact.org/events/3ie-conferences-and-workshops/mexico-impact-evaluation-conference/.

[6] Cartwright N (2011) Knowing what we are talking about: Why evidence doesn't always travel. Conference presentation 31 May. Available: http://www.3ieimpact.org/media/filer/2012/05/03/knowing_what_talking_31emay2011.pdf.

[7] Deaton A (January 2009) The Keynes Lecture - Randomization in the tropics, and the search for the elusive keys to economic development. Available: http://www.econ.uiuc.edu/~roger/courses/574/readings/Deaton_Instruments%20of%20development.pdf.

[8] Rosenzweig M (2012) Thinking Small: Poor Economics: A Radical Rethinking of the Way to Fight Global Poverty. J Econ Lit 50: 115–127.

[9] Rodrik D (2008) The New Development Economics: We Shall Experiment, But How Shalle We Learn? Available: http://www.hks.harvard.edu/fs/drodrik/Research%20papers/The%20New%20Development%20Economics.pdf.

[10] Ravallion M (2012) Fighting Poverty One Experiment at a Time: Poor Economics: A Radical Rethinking of the Way to Fight Global Poverty: Review Essay. J Econ Lit 50: 103–114.

[11] Murray CJ, Lopez AD (1996) Evidence-based health policy–lessons from the Global Burden of Disease Study. Science 274: 740–743. 8966556 10.1126/science.274.5288.740

[12] Tang KC, Choi BC, Beaglehole R (2008) Grading of evidence of the effectiveness of health promotion interventions. J Epidemiol Community Health 62: 832–834. 18701736 10.1136/jech.2007.061366

[13] Guyatt GH, Oxman AD, Vist GE, Kunz R, Falck-Ytter Y, et al (2008) GRADE: an emerging consensus on rating quality of evidence and strength of recommendations. BMJ 336: 924–926. 18436948 10.1136/bmj.39489.470347.AD PMC2335261

[14] Baral S, Wirtz A, Sifakis F, Johns B, Walker D, et al (2012) “The Highest Attainable Standard of Evidence (HASTE) for HIV/AIDS Interventions: Towards A Public Health Approach to Defining Evidence. Public Health Reports. 127: 572–584. 10.1177/003335491212700607 PMC3461350 23115382

[15] Briss PA, Zaza S, Pappaioanou M, Fielding J, Wright-De Aguero L, et al (2000) Developing an evidence-based Guide to Community Preventive Services–methods. The Task Force on Community Preventive Services. Am J Prev Med 18: 35–43. 10806978 10.1016/s0749-3797(99)00119-1

[16] National Health and Medical Research Council (December 2009) NHMRC levels of evidence and grades for recommendations for developers of guidelines. Australia. Available: http://www.nhmrc.gov.au/guidelines/resources-guideline-developers. Accessed June 2013.

[17] Wightman A, Ellis S, Cullum A, Sander L, Turley R (2005) Grading evidence and recommendations for public health interventions: developing and piloting a framework. Health Development Agency. Available: http://www.nice.org.uk/nicemedia/docs/grading_evidence.pdf. Accessed December 2012.

[18] Shekelle PG, Maglione MA, Luoto J, Johnsen B, Perry TR. Global Health Evidence Evaluation Framework. Research White Paper (Prepared by the Southern California Evidence-based Practice Center under Contract No. 290-2007-10062-I). AHRQ Publication No. 13-EHC008-EF. Rockville, MD: Agency for Healthcare Research and Quality. January 2013. Available: http://www.effectivehealthcare.ahrq.gov/ehc/products/363/1384/White-Paper_Global-Health-Frameworks-1-22-13.pdf 23427349

[19] Brunetti M, Shemilt I, Pregno S, Vale L, Oxman AD, et al (February 2013) Grade guidelines: 10. Considering resource use and rating the quality of economic evidence. J Clin Epidemiol 66: 140–150. 22863410 10.1016/j.jclinepi.2012.04.012

[20] Atkins D, Best D, Briss PA, Eccles M, Falck-Ytter Y, et al (2004) Grading quality of evidence and strength of recommendations. BMJ 328: 1490. 15205295 10.1136/bmj.328.7454.1490 PMC428525

[21] Berkman ND, Lohr KN, Morgan LC, Richmond E, Kuo TM, et al. (May 2012) Reliability Testing of the AHRQ EPC Approach to Grading the Strength of Evidence in Comparative Effectiveness Reviews. Methods Research Report. (Prepared by RTI International–University of North Carolina Evidence-based Practice Center under Contract No. 290-2007-10056-I.) Rockville, MD: Agency for Healthcare Research and Quality. AHRQ Publication No. 12-EHC067-EF. Available: http://effectivehealthcare.ahrq.gov/ehc/products/339/1099/Methods_ReliabilityTesting_FinalReport_20120522.pdf. 22764383

[22] Hartling L, Ospina M, Liang Y, Dryden DM, Hooton N, et al (2009) Risk of bias versus quality assessment of randomised controlled trials: cross sectional study. BMJ 339: b4012. 19841007 10.1136/bmj.b4012 PMC2764034

[23] Mohler R, Bartoszek G, Kopke S, Meyer G (2012) Proposed criteria for reporting the development and evaluation of complex interventions in healthcare (CReDECI): guideline development. Int J Nurs Stud 49: 40–46. 21924424 10.1016/j.ijnurstu.2011.08.003

[24] Shekelle PG, Pronovost PJ, Wachter RM, Taylor SL, Dy SM, et al (2011) Advancing the science of patient safety. Ann Intern Med 154: 693–696. 21576538 10.7326/0003-4819-154-10-201105170-00011

[25] Lewin S, Bosch-Capblanch X, Oliver S, Akl EA, Vist GE, et al (2012) Guidance for Evidence-Informed Policies about Health Systems: Assessing How Much Confidence to Place in the Research Evidence. PLoS Med 9: e1001187 doi:10.1371/journal.pmed.1001187 22448147 10.1371/journal.pmed.1001187 PMC3308931

[26] Arnold BF, Colford JM Jr (2007) Treating water with chlorine at point-of-use to improve water quality and reduce child diarrhea in developing countries: a systematic review and meta-analysis. Am J Trop Med Hyg 76: 354–364. 17297049

[27] Clasen T, Schmidt WP, Rabie T, Roberts I, Cairncross S (2007) Interventions to improve water quality for preventing diarrhoea: systematic review and meta-analysis. BMJ 334: 782. 17353208 10.1136/bmj.39118.489931.BE PMC1851994

[28] Siegfried N, van der Merwe L, Brocklehurst P, Sint TT (2011) Antiretrovirals for reducing the risk of mother-to-child transmission of HIV infection. Cochrane Database Syst Rev CD003510. 21735394 10.1002/14651858.CD003510.pub3 PMC13168846

[29] Johri M, Ako-Arrey D (2011) The cost-effectiveness of preventing mother-to-child transmission of HIV in low- and middle-income countries: systematic review. Cost Eff Resour Alloc 9: 3. 21306625 10.1186/1478-7547-9-3 PMC3045936

[30] Chigwedere P, Seage GR, Lee TH, Essex M (2008) Efficacy of antiretroviral drugs in reducing mother-to-child transmission of HIV in Africa: a meta-analysis of published clinical trials. AIDS Res Hum Retroviruses 24: 827–837. 18544018 10.1089/aid.2007.0291

[31] Lewin S, Munabi-Babigumira S, Glenton C, Daniels K, Bosch-Capblanch X, et al (2010) Lay health workers in primary and community health care for maternal and child health and the management of infectious diseases. Cochrane Database Syst Rev CD004015. 20238326 10.1002/14651858.CD004015.pub3 PMC6485809

