# Supplementary material for: A Comparison of Frameworks Evaluating Evidence for Global Health Interventions
Source: PLoS Med. 2013 Jul 9;10(7):e1001469. doi: 10.1371/journal.pmed.1001469 (PMC3706307; doi:10.1371/journal.pmed.1001469)
Supplement: Box S1 — Search methodology. (DOC) [file pmed.1001469.s001.doc]

**Supporting Information Box S1:**

We identified existing frameworks for use in public health or global health interventions by searching Medline, web sites, and contacting experts. We searched Medline up until March 2012, using terms such as “public health intervention,” “strength of evidence,” and “quality of evidence,” and as well as “criteria,” rating,” system” etc. The vast majority of the results were reviews of the effectiveness of public health interventions (they used the terms in their texts), rather than actual systems to rate the evidence. We reviewed the web sites of the Cochrane Collaboration, the Campbell Collaboration, the SUPporting POlicy relevant Reviews and Trials (SUPPORT) project*,* the International Initiative for Impact Evaluation (3IE), the World Health Organizations’ EVIPnet (Evidence Informed Policy Networks), SURE (Supporting the Use of Research Evidence for policy in African health systems) and the McMaster Health Forum. We also received input from our experts to identify global health evidence frameworks.
