# Supplementary material for: A Comparison of Frameworks Evaluating Evidence for Global Health Interventions
Source: PLoS Med. 2013 Jul 9;10(7):e1001469. doi: 10.1371/journal.pmed.1001469 (PMC3706307; doi:10.1371/journal.pmed.1001469)
Supplement: Box S2 — Full list of potential exemplars. (DOC) [file pmed.1001469.s002.doc]

**Supporting Information Box S2:**

The set of global health exemplars originally considered for inclusion were:

- Malaria bednets
- A malaria vaccine
- The prevention of the mother-to-child transmission of HIV (selected)
- Household water chlorination (selected)
- Free condom distribution
- Treatment for HIV as prevention of the spread of infection
- Oral rehydration salts for acute diarrhea
- Handwashing interventions to prevent the spread of infectious disease
- Improved cook stove adoption to prevent acute respiratory illness
- (Village-level) source water improvement.
- Our third selected exemplar of community health workers to reduce child mortality was not on our original list but was selected in consultation with our TEP.
