# Supplementary material for: A Comparison of Frameworks Evaluating Evidence for Global Health Interventions
Source: PLoS Med. 2013 Jul 9;10(7):e1001469. doi: 10.1371/journal.pmed.1001469 (PMC3706307; doi:10.1371/journal.pmed.1001469)
Supplement: Table S1 — Diversity of exemplar interventions across key dimensions. (DOC) [file pmed.1001469.s003.doc]

**Table S1. Diversity of Exemplar Interventions across Key Dimensions**

| **Dimension / Examples** | **Household water chlorination** | **Preventing Mother-to-Child Transmission for HIV** | **Lay health workers in primary or community health care to reduce mortality and morbidity** |
| --- | --- | --- | --- |
| **Populations affected by a disease** | | | |
| - maternal health, adults, elderly, children | primarily children under 5 (diarrhea) | pregnant women | all, primarily children under 5 |
| - Africa vs. Asia vs. Latin America | all | Sub-Saharan Africa, Southeast Asia | all, primarily children under 5 |
| **Communicable vs. non-communicable** | communicable | communicable | Both |
| **One time vs. continuous behavior** | continuous | time limited | Both |
| **Preventive or treatment** | prevent | prevent | prevent and treat |
| **Publicly provided or rely on private decisions/marketplaces vs. is this deliverable by the health system** | could be either | often publicly provided by NGOs | generally publicly provided by health system |
| **Resource dimension** | Low | high | unknown |
| **Individual vs. Community - level of impact** | equally individual | mostly individual | mostly individual |
| **Individual vs. Community - level of intervention** | individual | individual | community |
| **Urban vs. Rural** | urban and rural | urban and rural | mostly rural |

Notes to table S1: The dimensions for classifying interventions included the following: the primary population affected by the disease or the intervention – both in terms of geography as well as demographics (e.g., pregnant women, children under five, Sub-Saharan Africa, etc.); whether the intervention addresses a communicable or non-communicable disease; whether the intervention necessitates a one-time delivery or demands a sustained change in behavior or practice on the part of one or more actors; whether the intervention is preventive or a treatment; the usual source for the delivery of the intervention – whether the health system, a non-governmental organization (NGO) or private actors; a broad approximation of the level of resources required to supply the intervention (categorized as ‘low’ or ‘high’); whether the intervention is delivered at the individual or community level (e.g., malaria bednets versus community boreholes); and whether the anticipated health benefits of the intervention are at the individual or community level.
