# Supplementary material for: A Comparison of Frameworks Evaluating Evidence for Global Health Interventions
Source: PLoS Med. 2013 Jul 9;10(7):e1001469. doi: 10.1371/journal.pmed.1001469 (PMC3706307; doi:10.1371/journal.pmed.1001469)
Supplement: Table S2 — Evidence base for three global health exemplars. (DOC) [file pmed.1001469.s004.doc]

**Table S2.Evidence base for 3 global health exemplars**

| **Exemplar** | **Existing Reviews** | **Included Studies** | **Setting** | **Findings** |
| --- | --- | --- | --- | --- |
| **Household water chlorination** | Arnold, B.F. et al., 2007 | Arnold: 10 studies of various designs with “adequately designed” control groups; | South Africa, Africa, Southeast Asia, Central Asia | Arnold Diarrheal Outcome - RR = 0.71 (95% CI: 0.58-0.87); |
|  | Clasen, T., et al., 2007 | Clasen: 16 RCTs and quasi RCTs, 4 included in meta-analysis for RR outcomes. |  | For Clasen: RR=0.41 (CI: 0.26 to 0.65) in diarrhea for all ages; RR=0.60 (CI: 0.41 to 0.87) in under 5s |
| **Preventing Mother-to-Child Transmission for HIV** | Siegfried, N., et al.,2011 | 25 RCTs | Africa | - Efficacy up to 60%, depending on regimen |
|  | Johri, M., et al., 2011 | 19 cost-effectiveness analyses | Low & Middle Income Countries (LMICs) | - 16 of 19 studies showed cost-effectiveness |
|  | Chigwedere, P., et al., 2008 | 10 RCTs | LMICs | - 50% reduction in MCTC |
| **Lay health workers in primary or community health care to reduce mortality and morbidity** | Lewin, S., et al., 2010 | 7 RCTs | Bangladesh, Thailand, Burkina Faso, India, Nepal, Vietnam | Reduction in morbidity and mortality in children less than 5 years old RR = 0.86 (95% CI: 0.75-0.99) |

Notes to Table S2: The literature on household water chlorination had two potential primary outcome measures: water quality (often objectively measured via water testing) and diarrheal outcomes, which are predominantly self-reported (often by the mother in the case of children’s diarrheal outcomes) but are often the primary outcomes reported in trials of chlorination studies. For the PMTCT exemplar, we used the rate of HIV infection in children, assessed at any time during the first year of life as the primary outcome. For Community or Lay Health Workers (CHWs) in developing countries, we adopted the literature’s convention of assessing its effectiveness according to the outcome of under-5 morbidity and mortality from any cause.
